# Presence of a functional but dispensable Nuclear Export Signal in the HTLV-2 Tax protein

**DOI:** 10.1186/1742-4690-2-70

**Published:** 2005-11-14

**Authors:** Sébastien A Chevalier, Laurent Meertens, Sara Calattini, Antoine Gessain, Lars Kiemer, Renaud Mahieux

**Affiliations:** 1 Unité d'Epidémiologie et Physiopathologie des Virus Oncogènes, Institut Pasteur, Paris, France; 2 Center for Biological Sequence Analysis, BioCentrum-DTU, The Technical University of Denmark, Building 208 DK-2800, Lyngby, Denmark

## Abstract

**Background:**

Human T-cell leukemia virus type 1 and type 2 are related human retroviruses. HTLV-1 is the etiological agent of the Adult T-cell Leukemia/Lymphoma and of the Tropical Spastic Paraparesis/HTLV-1 Associated Myelopathy, whereas, HTLV-2 infection has not been formally associated with any T-cell malignancy. HTLV-1 and 2 genomes encode, respectively, the Tax1 and Tax2 proteins whose role is to transactivate the viral promoter. HTLV-1 and HTLV-2 Tax sequences display 28% divergence at the amino acid level. Tax1 is a shuttling protein that possesses both a non canonical nuclear import (NLS) and a nuclear export (NES) signal. We have recently demonstrated that Tax1 and Tax2 display different subcellular localization and that residues 90–100 are critical for this process. We investigate in the present report, whether Tax2 also possesses a functional NES.

**Results:**

We first used a NES prediction method to determine whether the Tax2 protein might contain a NES and the results do suggest the presence of a NES sequence in Tax2. Using Green Fluorescent Protein-NES (GFP-NES) fusion proteins, we demonstrate that the Tax2 sequence encompasses a functional NES (NES2). As shown by microscope imaging, NES2 is able to mediate translocation of GFP from the nucleus, without the context of a full length Tax protein. Furthermore, point mutations or leptomycin B treatment abrogate NES2 function. However, within the context of full length Tax2, similar point mutations in the NES2 leucine rich stretch do not modify Tax2 localization. Finally, we also show that Tax1 NES function is dependent upon the positioning of the nuclear export signal "vis-à-vis" GFP.

**Conclusion:**

HTLV-2 Tax NES is functional but dispensable for the protein localization *in vitro*.

## Background

HTLV-1 and HTLV-2 are closely related retroviruses that infect T-cells *in vivo*, with a probable preferential tropism for CD4^+ ^and CD8^+ ^cells respectively [1]. HTLV-1 is the etiological agent of the Adult T-cell Leukemia/Lymphoma (ATLL) and of the Tropical Spastic Paraparesis/HTLV-1 Associated Myelopathy (TSP/HAM), while HTLV-2 infection, even if originally described in a patient suffering of atypical hairy T-cell leukemia, has only been linked to infrequent cases of TSP/HAM "like" disease [2-4]. Both HTLV-1 and HTLV-2 genomes encode a viral transactivator (Tax1 and Tax2 respectively). Tax1 has an oncogenic potential and is responsible for cell-transformation *in vitro *[5,6]. Tax1 and Tax2 display approximately 75% nucleotide sequence homology. Strikingly however, several reports have now demonstrated that although the critical functional regions of the proteins are well conserved (i.e. NF-κB and CREB/ATF activation domains), the two transactivators exhibit a number of major phenotypical differences [1,7-18]. Nevertheless, Tax2 is capable of immortalizing human lymphocytes and, although to a lesser extent than Tax1, of transforming rat cells *in vitro *[10,19].

Eukaryotic cells are compartmentalized into the cytoplasm and the nucleus by the nuclear envelope [20,21]. The nuclear envelope contains nuclear pore complexes (NPCs), which mediate the traffic of molecules between the two compartments. The nucleo-cytoplasmic traffic of large molecules is regulated by specific nuclear import and export systems. Proteins that contain classical Nuclear Localization Signals (NLSs) are imported into the nucleus by importin α/β protein heterodimers. So far, six importin α family members and one importin β have been described [22]. Importin α binds to NLS containing proteins, whereupon importin β is responsible for the docking of the importin cargo complex to the cytoplasmic side of the NPC, followed by translocation of the complex through the NPC. A classical monopartite NLS consists of a stretch of basic amino acids such as arginines and lysines. Contrary to this, the Nuclear Export Signal (NES) generally consists of a leucine/isoleucine-rich sequence [23]. The classical NES pattern is L-x(2,3)- [LIVFM]-x(2,3)-L-x- [LI], where L can either be L, I, V, F or M, but many known NES regions do not conform to these limitations [24]. For example, the spacing between the hydrophobic residues is variable and NES regions can also be rich in glutamate, aspartate and serine [23]. The first nuclear export pathway to be discovered involved the chromosome region maintenance 1 (CRM1) receptor, exporting proteins containing a nuclear export signal (NES) [25]. CRM1 binds to a Nuclear Export Signal (NES)-containing protein and to the NPC. Several ways of regulating NES-dependent export have been reported, including masking or unmasking the NES and post-translational modifications of the NES-containing protein [26].

Cellular fractionation and immunofluorescence experiments performed with HTLV-1 infected and Tax1 transfected cells have demonstrated that Tax1 was present both in the nuclear and cytoplasmic fractions. However, the distribution of the protein between these two compartments is unequal and depends on the cell-line used [27-31]. The Tax1 48 amino terminal sequence contains a non-canonical functional NLS [32] that allows the protein to enter the nucleus, where Tax1 localizes to discrete nuclear bodies (also called Tax Speckled Structures (TSS) [33]. In addition, Tax1 also contains a "Rev-like" Nuclear Export Signal (NES) spanning from amino acid 189 to 202. This NES is insensitive to leptomycin B within the context of the full-length protein [27]. Both localization signals (NLS and NES) are likely to be involved in the shuttling of Tax1, but this process is still not clearly understood [34].

We have recently reported that, although Tax2 contains a functional NLS domain, the protein localizes predominantly to the cytoplasm in HTLV-2 immortalized or transformed infected T-cells as well as in Tax2 transfected cells [16]. These results were further confirmed in another laboratory [35] which also demonstrated that the NLS domain was confined to the 40 first N-terminal amino acids. We also demonstrated that the region spanning amino acids 90 to 100 was critical for Tax2 localization [16]. The recent report of a Tax1 NES sequence prompted us to examine the possible presence of a NES in Tax2. In addition to the 90–100 domain, this sequence could serve as a second domain involved in Tax2 localization. We show in this report that, although HTLV-2 Tax protein contains a NES sequence that is active without the context of a full-length protein, this domain is dispensable for the Tax2 localization.

## Results

### HTLV-2 Tax protein sequence contains a putative NES domain

We lately demonstrated that the HTLV-2 Tax protein has an intracellular localization that is different from that of Tax1, both in infected and transfected cells (i.e. Tax2 localizes more to the cytoplasm than Tax1) and that, within the Tax sequence, the 90–100 domain was critical for the protein localization [16]. These results were confirmed lately [35]. Another recent article reported that, in addition to the previously characterized NLS, HTLV-1 Tax protein also contains a Nuclear Export Signal (NES) comprising amino acids 189 to 202 (KRIEELLYKISLTT). This sequence contains a string of hydrophobic amino acids (I191, L195, I198 and L200) [27] and has the ability to redirect the Green Fluorescent Protein (GFP) to the cytoplasm. Within the Tax1 NES sequence, residues L195 (formerly named L194 [27]) and L200 appear to be critical for the Tax1 NES function. As an example, when Tax1 L200 is mutated to an alanine, the GFP-Tax1 localization is altered [27].

**Figure 1:**
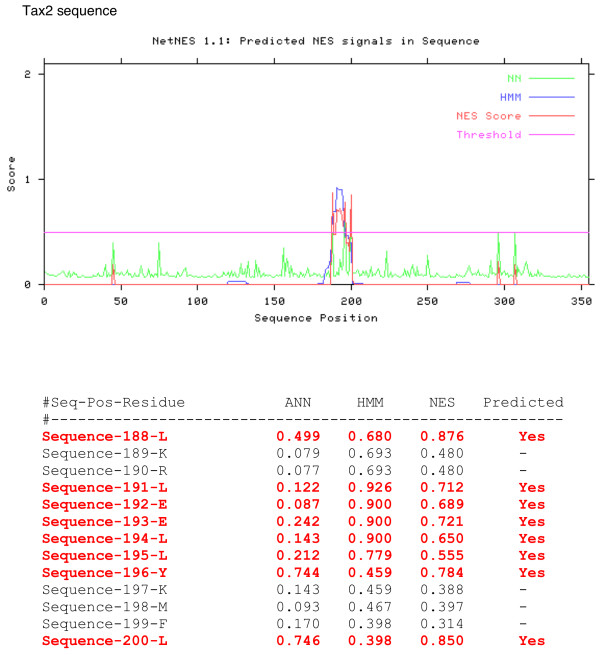
Output after submission of the complete Tax2 amino acid sequence to the NetNES website [23]. Output consists of two parts; one is a listing of all residues with the individual scores noted (see column heading), the other is a graphical plot of the values given in the table. The prediction server calculates the NES score from the HMM and Artificial Neural Network (ANN) scores but all three values are given for each residue. If the calculated 'NES score' exceeds the threshold, then that particular residue is expected to participate in a nuclear export signal. This is denoted with a 'Yes' in the column "Predicted".

**Figure 2:**
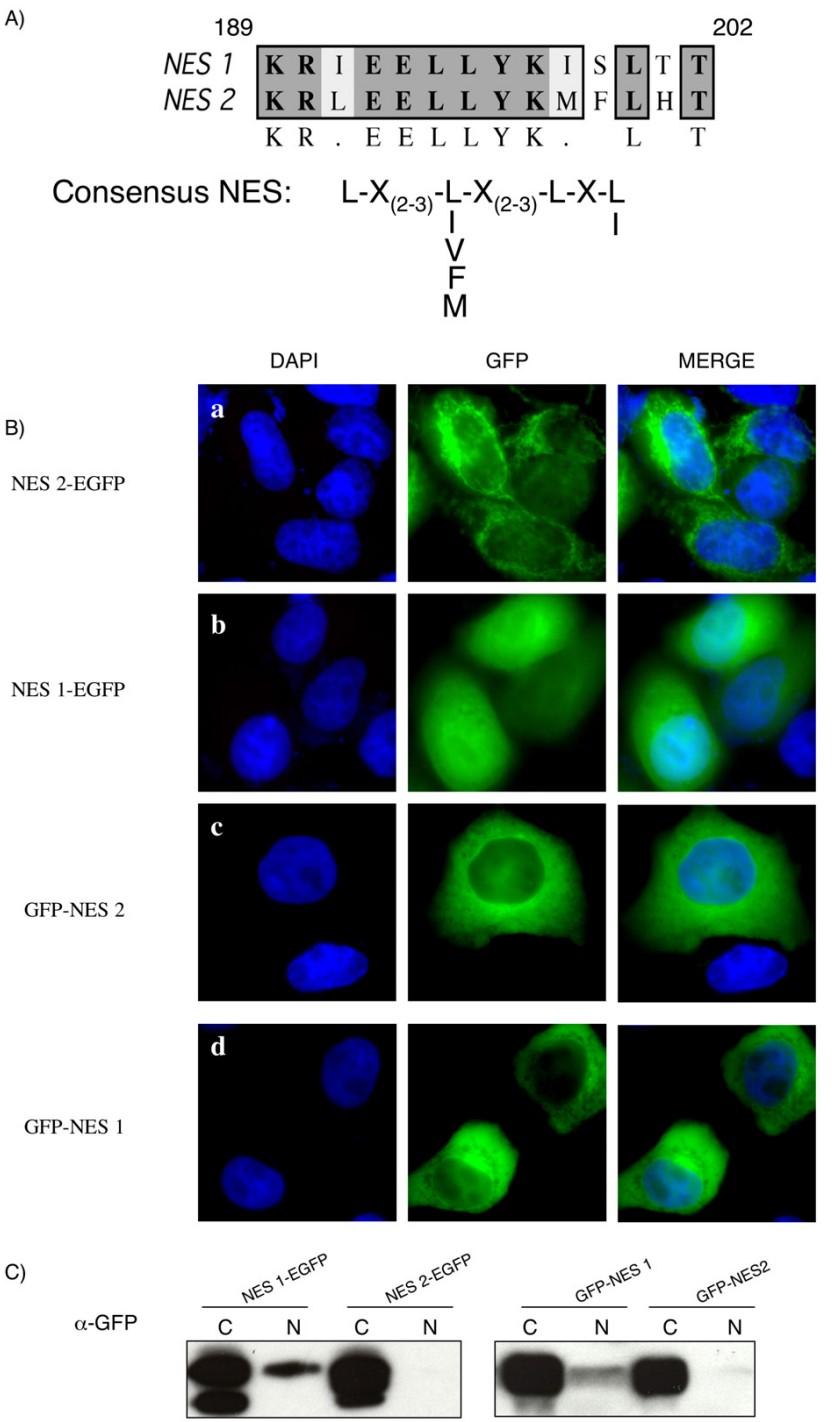
Without the context of the full length protein, NES2 can redirect GFP to the cytoplasm, while NES1 function depends on its positioning vis-à-vis GFP. (A): Sequence alignment of a consensus NES sequence with Tax1 NES and Tax2 putative NES. (B): HeLa cells were transiently transfected with NES-EGFP and GFP-NES plasmids. Twenty-four hours after transfection, the cells were washed with PBS, fixed with 4% paraformaldehyde, mounted with DAPI-containing mounting medium and visualized with a Zeiss Axioplan 2 imaging microscope X40 using a Zeiss Axiocam HRc (color) camera and the Zeiss Apotome software. Images of cells that are representative of the entire population are shown. (C): Western-blot analysis of cytoplasmic and nuclear cell fractions. 293T cell fractions were subjected to electrophoresis on a 10 % TG gel and probed with a GFP antibody. The western-blot results are representative of four independent experiments.

In order to identify whether Tax2 contains a similar sequence, we first used the NetNES prediction method [23]. (Figure 1). From this analysis, residue L188 of Tax2 was predicted to be part of the Tax2 NES domain. Interestingly, L188 is absent from the sequence of Tax1, where the position is occupied by a tyrosine (data not shown). The amino acid comparison of Tax1 and Tax2 also reveals that the two sequences are very similar in the 189 to 202 amino acids region, with observed differences at positions 191, 198, 199 and 201 (Figure 2A).

We set out to investigate whether, despite these differences, the Tax2 putative NES was functional. To this end, we affixed the 189–202 amino-acid domain of Tax2 to the N-terminus of the GFP sequence (NES2-EGFP) using the pEGFP-N1 vector as previously reported [27]. The NES2-EGFP construct was then transiently transfected in 293T (data not shown) and Hela cells, as these cells have frequently been used for Tax localization studies [17,27]. As a positive control, the NES Tax1 sequence was also fused to the N-terminus of the GFP (NES1-EGFP). In the absence of a Tax NES sequence, the GFP protein is nearly equally distributed between the cytoplasm and the nucleus of the transfected cells ([16] and data not shown). However, the GFP signal was almost entirely cytoplasmic when the protein was fused to the Tax2 putative NES (Figure 2B panel a and Figure 2C for fractionation). This suggests that this latter sequence mediates an active transport of GFP *in vitro*. Unexpectedly, and contrary to a previous report [27], the NES1-EGFP fusion protein was diffused in both the nucleus and the cytoplasm with a nuclear content that was much higher than that of NES2-EGFP (Figure 2B panels a vs. b and Figure 2C). We obtained and sequenced the construct that has been used in Dr Wigdahl's laboratory and the sequence results showed that the Tax1 NES domain had been cloned to the C-terminus part of the GFP rather than to the N-terminus (data not shown). Consequently, a second series of recombinant plasmids was made using the pGFP-C3 vector, allowing for a GFP C-terminal fusion construct. As with the NES2-EGFP construct, GFP-NES2 was mostly cytoplasmic (Figure 2B panel c), while, under these experimental conditions, GFP-NES1 was also, as previously published, preponderant in the cytoplasm (Figure 2B panel d). Interestingly, subcellular fractionation experiments clearly demonstrated that, even in that case, the GFP-NES1 nuclear fraction was more abundant than that of GFP-NES2 (Figure 2C right panel). Altogether, these results suggest that, without the context of a full length protein, Tax2 NES domain is active both when fused to the N- or to the C-terminus part of the GFP, while Tax1 NES functions more efficiently when fused to the C-end of GFP.

### Within Tax2 NES sequence, several leucine residues are critical for a CRM-1 dependent function

**Figure 3:**
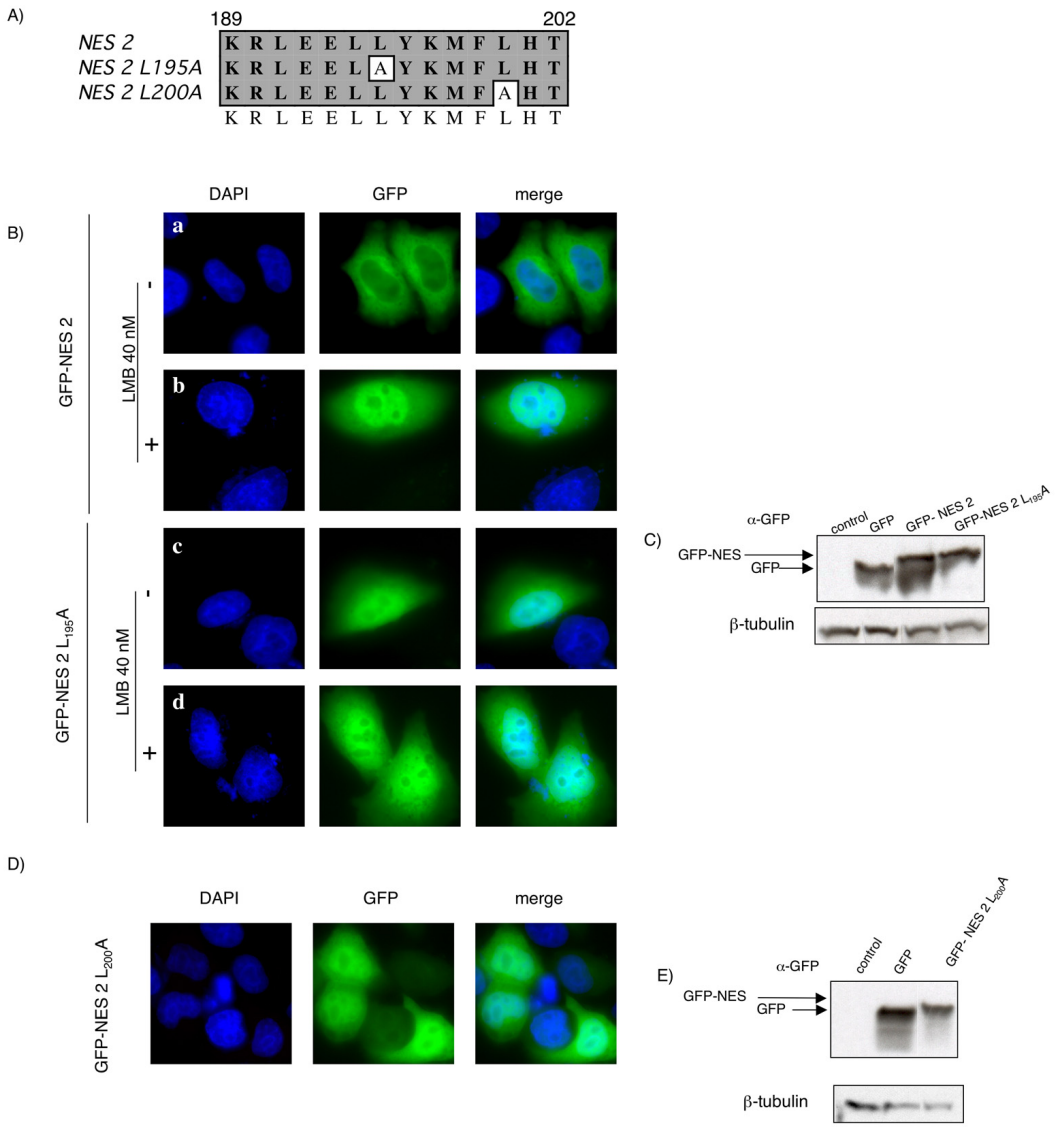
Nucleocytoplasmic distribution of GFP-NES2 is altered after incubation with leptomycin B or by single point mutations. (A): Sequence alignment of wild-type Tax2 and Tax2 GFP-NES mutants. (B and D): HeLa cells were transiently transfected with the different GFP-NES plasmids. Eighteen hours post transfection, transfected cells were treated with leptomycin B (40 nM) or methanol for 3 hours. Cells were then washed, fixed, mounted with DAPI-containing medium and visualized with a Zeiss Axioplan 2 imaging microscope X40 using a Zeiss Axiocam HRc (color) camera and the Zeiss Apotome software. Images of cells that are representative of the entire population are shown. (C and E): Western-blot analysis of GFP and GFP-NES proteins. 293T cell lysates (70 μg) were subjected to electrophoresis on a 10 % TG gel and probed with GFP or β-tubulin antibodies.

We next investigated whether Tax2 NES activity was dependent upon the CRM-1 pathway. To this end, Hela cells were transfected with the different NES constructs (Figure 3A), with or without leptomycin B (LMB). LMB blocks CRM1-dependent nuclear export and has been used extensively to probe this process [36]. In the presence of LMB, GFP-NES2 localizes to the nucleus, suggesting that the CRM-1 pathway is involved in the shuttling of the fusion protein (Figure 3B panel b). As a control, incubation of the GFP-NES transfected cells with methanol (the solvent which has been used to dissolve LMB in panel b), had no effect (Figure 3B panel a). Leucine 195 (formerly named 194 [27]) has been shown to be critical for the NES1 ability to export the GFP protein via the CRM-1 dependant pathway. Since the sequence of NES2 also contains a leucine at position 195, we mutated this residue to an alanine. This mutation abrogated the nuclear export of GFP-NES2 (Figure 3B, panel c). As expected, adding LMB to the GFP-NES2 L_195_A had no effect on the protein localization (Figure 3B, panel d). Mutating leucine 200 to an alanine also suppressed NES function (Figure 3D), while the L_194_A mutation had no effect (data not shown). Altogether these results confirm that, within the Tax2 NES domain, more than one leucine residues are needed for the function of the export signal. Western blot controls show that the overall protein expression was comparable between the different constructs (Figures 3C and 3E).

### Evaluating the role of Tax2 leucine 188

**Figure 4:**
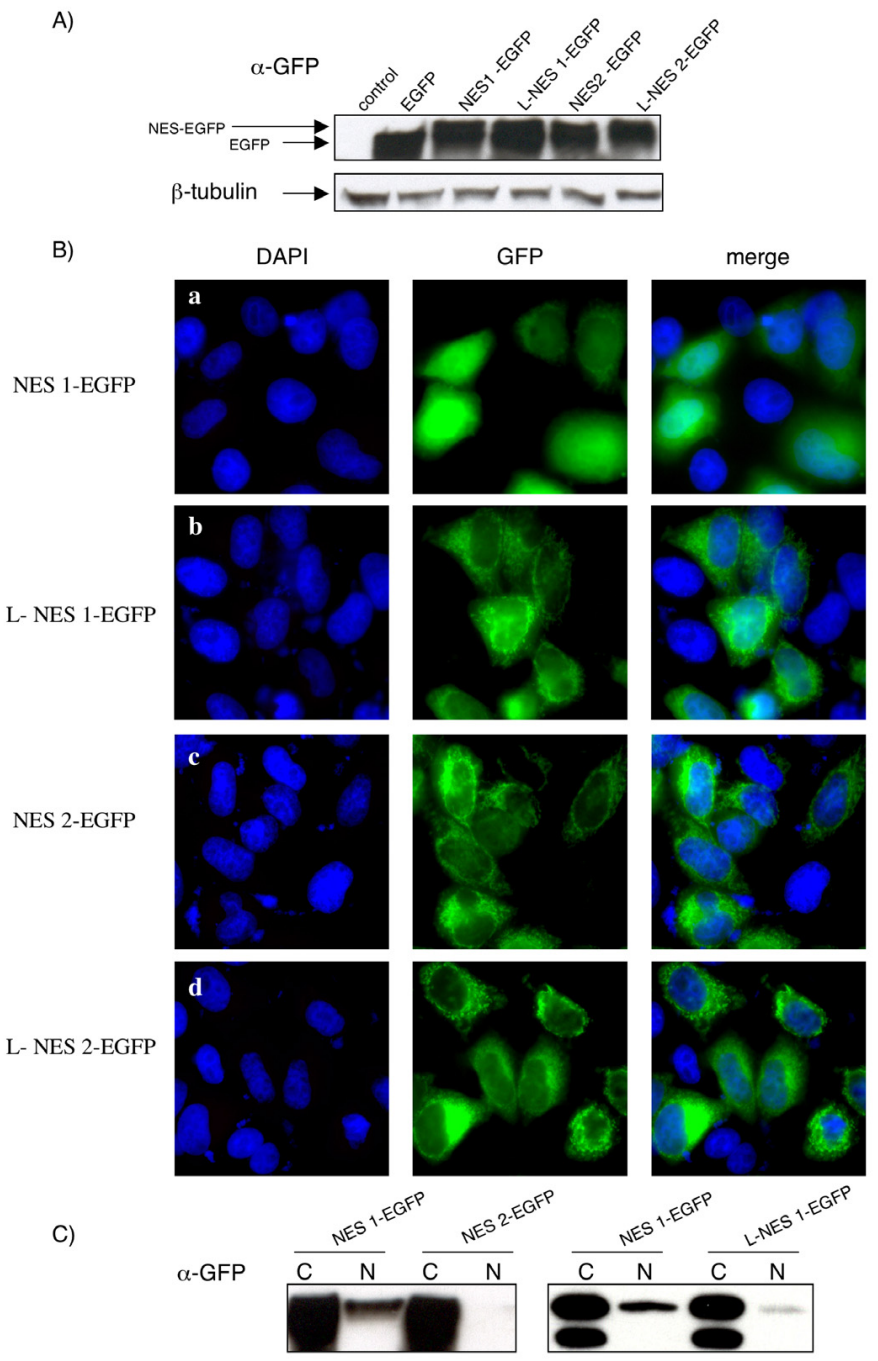
The presence of leucine 188 restores NES1 function within the context of a NES1-EGFP protein. (A): Western-blot analysis of the different EGFP and NES-EGFP proteins. 293T cell lysates (70 μg) were subjected to electrophoresis on a 10 % TG gel and probed with GFP or β-tubulin antibodies. (B): HeLa cells were transiently transfected with the different NES-EGFP plasmids. Eighteen hours post transfection, transfected cells were washed, fixed, mounted with DAPI-containing medium and visualized with a Zeiss Axioplan 2 imaging microscope X40 using a Zeiss Axiocam HRc (color) camera and the Zeiss Apotome software. Images of cells that are representative of the entire population are shown. (C): 293T nuclear and cytoplasmic cell fractions were subjected to electrophoresis on a 10 % TG gel and probed with a GFP antibody. The western-blot results are representative of four independent experiments.

The NES prediction software results suggested that L188 might be part of NES2 (Figure 1). To evaluate the role of this amino acid in Tax2 NES function we constructed another series of NES-EGFP plasmids, in which amino acid leucine #188 was added to the autologous (i.e. NES2) or to the heterologous NES (i.e. NES1) sequences. The constructs were transfected into Hela cells and the expression of the fusion proteins determined by western-blot (Figure 4A). As described above, the NES1-EGFP has a stronger nuclear localization than NES2-EGFP (Figure 4B panels a vs. c). This is correlated with the fractionation experiment (Figure 4C left panel). Remarkably, adding a leucine to the NES1-EGFP sequence improved the "NES" phenotype, since the nuclear fraction is lower in the presence of leucine 188 (Figure 4B panel a vs. b and Figure 4C right panel). Adding leucine 188 to the _189_NES2_202_-EGFP sequence increased only modestly the percentage of cells in which the signal was cytoplasmic (Figure 4B, panels c vs. d). Altogether, these results demonstrate that a leucine residue at position 188 allows a better export of the NES1-EGFP fusion protein. However, this leucine is dispensable in the context of a GFP-NES1 protein.

### The localization of GFP-Tax2 is not altered by mutations in the NES

**Figure 5:**
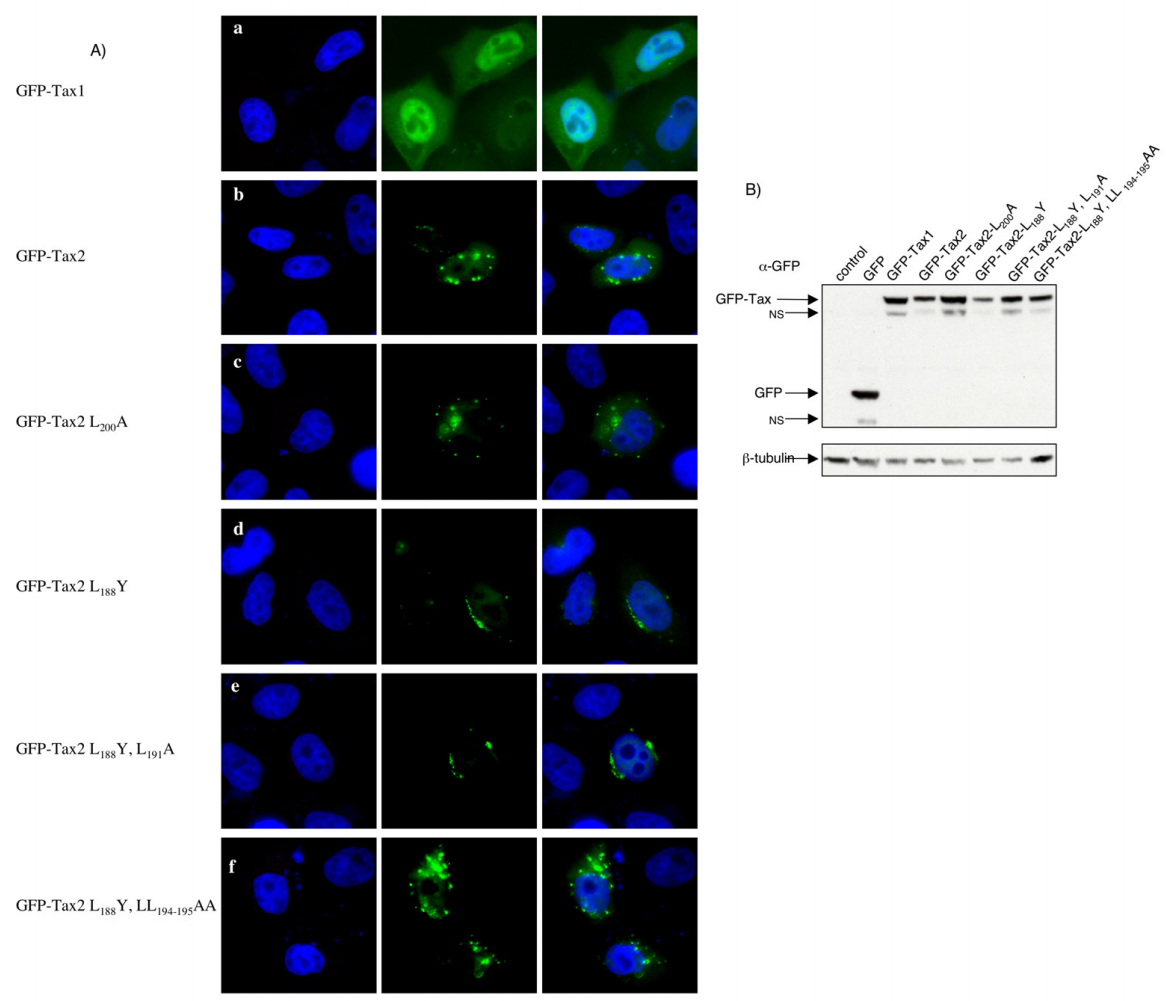
Within the context of a full length Tax2 protein, the presence of a functional NES2 domain is dispensable for the protein localization. (A): HeLa cells were transiently transfected with the GFP-Tax1, GFP-Tax2 and the different GFP-Tax2 mutants plasmids. Eighteen hours post transfection, the cells were washed, fixed, mounted with DAPI-containing medium and visualized with a Zeiss Axioplan 2 imaging microscope X40 using a Zeiss Axiocam HRc (color) camera and the Zeiss Apotome software. Images of cells that are representative of the entire population are shown. (B): Western-blot analysis of GFP and GFP-NES proteins. 293T cell lysates (70 μg) were subjected to electrophoresis on a 10 % TG gel and probed with GFP or β-tubulin antibodies.

Although the results presented above have shown that the Tax2 NES represents an active domain in the context of the NES2-EGFP and GFP-NES2 chimera proteins, it was important to examine the function of the NES2 within the context of the full-length Tax2 protein. To do this, point mutations were made in the GFP-Tax2 full-length construct, with one or several leucine residues (up to three) mutated within the NES2. We also mutated residue 188 in order to evaluate the role of this amino-acid in the context of the complete protein. Unexpectedly, all these mutated Tax proteins, i.e. GFP-Tax2 L_188_Y, GFP-Tax2 L_188_Y, L_191_A, GFP-Tax2 L_188_Y, LL_194–195_AA, GFP-Tax2 L_200_A, had a predominant cytoplasmic localization and behaved mostly like Tax2 wild-type, i.e. with a strong cytoplasmic localization (Figure 5A, panels c, d, e, f as compared to b), but not like Tax1 (Figure 5A, panel a). Western-blot analysis demonstrated a comparable level of protein expression (Figure 5B). These results suggest that the presence of a wild-type NES2 is dispensable for exiting the cell nucleus in the context of the full-length Tax2 protein. As a control, we also used a GFP-Tax1 L_200_A vector. Strikingly however, in our hands this protein had a localization that was very similar to that of wild-type Tax-1 i.e. strong nuclear signal (data not shown). Indeed, we did not observe a strong localization to the nuclear membrane as it has been previously described. However, we should point out that we have used a GFP-Tax1 construct, while Alefantis et al used a Tax1-EGFP [27]. We cannot rule out the fact that the positioning of Tax1 L_200_A vis-à-vis GFP plays a role in the protein localization, although this is unlikely, since we previously observed that the localization of GFP-Tax1 was similar to that of Tax1-GFP [16].

## Discussion

Both in infected and in transfected cells, Tax1 and Tax2 are found in the nucleus and in the cytoplasm in different proportions: Tax1 being more abundant in the nucleus, while Tax2 is more prone to be found in the cytoplasm [16,35]. In the nucleus, Tax1 and Tax2 interact with transcription factors and activate the cyclic-AMP response element and activating transcription factor (ATF) binding (CREB/ATF) pathway, while in the cytoplasm the viral transactivators interact with several members of the NF-κB transduction pathway [5,37]. Tax1/Tax2 activation of CREB/ATF is needed for an efficient viral gene expression, while the permanent activation of NF-κB has been suggested to be critical, at least in HTLV-1 infected cells, for evading apoptosis. In order to activate the CREB/ATF and NF-κB pathways, both Tax1 and Tax2 must therefore shuttle between these two compartments [34].

A Nuclear Export Signal (amino acid 189 to 202) has recently been described in Tax1 [27]. Amino acids 1 to 58 constitute non canonical Nuclear Localization Signals [16,32] in both Tax1 and Tax2, but amino acids 90 to 100 are also critical for the localization of the viral transactivators [16]. Using prediction software as well as *in vitro *assays, we now describe another domain of Tax2. This sequence represents a Nuclear Export Signal (NES), with different functional characteristics from that of NES1. For example, the percentage of the GFP-NES2 protein that is present in the nucleus of the transfected cells is slightly different from that of GFP-NES1 protein. In addition, Tax2 NES is functional, no matter if it is fused to the N-terminal or the C-terminal of GFP, which is not the case of NES1 which is more active when fused to the C-terminus of GFP. We have also determined here that the NES of Tax2 can direct nuclear export via the CRM1 pathway, and that point mutations at positions 195 and 200 abrogate NES mediated translocation. All in all, these results demonstrate that the NES sequences of Tax1 and Tax2 have different functional profiles reflecting their slightly different sequences, and that the divergent amino acids are likely to be critical for the NES activity. The predictor software suggested that, in the Tax2 sequence, leucine 188 might also be part of the NES domain. This leucine is absent from Tax1 and, strikingly, when added to the NES1-EGFP construct, it restores the function of the Tax1 NES.

However, the most important result of this study is that, within the context of the whole Tax2 protein, mutating one or several leucine residues has no or an extremely limited impact on Tax2 localization. This could have been indicative of a secondary NES in the sequence being able to mediate translocation on its own, but this theory is not supported by the NetNES computational analysis. Therefore, this hypothesis is very unlikely. It would also disagree with our report that LMB treatment of Tax2 transfected cells did not abolish protein translocation [16]. Hence, we consider that the very modest increase in the GFP-Tax2 nuclear signal observed with some GFP-Tax2 mutants constructs as compared to GFP-Tax2 is not consistent with a strong use of this NES sequence by Tax2. Altogether, these results imply that the Tax2 protein uses other means of export from the cell nucleus leading to the observed strong cytoplasmic signal. This is consistent with our previous results showing that the 90–100 Tax domain, which does not behave as a NES, is critical for the protein localization [16]. Our results are therefore paradoxical: while Tax1 possesses a NES domain, it localizes predominantly in the nucleus at the equilibrium, whereas Tax-2, whose NES sequence is dispensable, has a predominant cytoplasmic localization.

In conclusion, without the context of the protein, both Tax1 and Tax2 seem to possess working nuclear export signals. If one regards the nuclear localization signal and nuclear export signal as competing forces, the Tax2 NES seems to be a more efficient mediator than that of Tax1 in terms of cytoplasmic versus nuclear abundance of the proteins without the context of a full-length protein. This observation is supported by the computational analysis as well as by our *in vitro *data. However, Tax2 does not need its NES signal to relocate to the cytoplasm. Rather, it seems to employ a different, hitherto uncharacterized translocation system, as we have previously suggested [16]. The implications of this paradox are that, even though a fully functional nuclear export signal is embedded in the Tax2 sequence, it is not actually necessary for the protein translocation under the conditions tested here. However, its functional conservation suggests that it might have a biological impact on the protein functions. Future *in vivo *studies will decipher whether the presence of the "NES" sequence in the HTLV-2 Tax protein has any role during the viral cycle.

## Methods

### Cell culture and drug treatment

Hela and 293T cells were grown in Dulbecco's modified Eagle's medium supplemented with 10% fetal bovine serum and antibiotics (penicillin 100 U/ml and streptomycin at 100 μg/ml). Cell lines were maintained at 37°C in 5% CO_2_. When indicated, cells were incubated with leptomycin B (Sigma) at 40 nM for 3 h.

### GFP-NES, NES-EGFP and GFP-Tax protein construction

The GFP-NES and NES-EGFP recombinants plasmids were obtained by cloning double stranded oligonucleotides into GFP-C3 and EGFP-N1 vectors (Clontech), using SacI/EcoRI and XhoI/PstI restriction sites respectively. Single or combined point mutations (at amino acids 188, 191, 194, 195 and 200) were also made in GFP-Tax1 and GFP-Tax2 sequences using the quick change mutagenesis kit (Stratagene) [16]. The nucleotide sequences of all constructs were determined using the DYEnamic ET Terminator Cycle Sequencing Kit (Amersham Biosciences) on an Applied Biosystems 373A DNA sequencer. Of note, during the course of these experiments, we noticed that the amino-acid numbering that has been used in Alefantis article was incorrect [27]. The first lysine of the Tax1 NES sequence is at position 189 and not 188 as reported previously. We have therefore modified the amino acid number accordingly.

### Transient transfection

For microscopic analyses, Hela cells were seeded in eight-well chamber glass slides, at a concentration of 3 × 10^4 ^cells/well and transfected the next day with 0,3 μg of DNA using the Effectene reagent (Qiagen). For immunoblot analyses, 293T cells were seeded on 6-well plates at 6 × 10^5 ^cells/well and transfected the next day with 2 μg of DNA using the Polyfect reagent (Qiagen) following the manufacturer's instructions.

### Immunoblot analyses

Twenty-four hours after transfection, 293T cells were washed twice with PBS, lysed (Tris-HCl pH 7,4 50 mM, NaCl 120 mM, EDTA 5 mM, NP40 0,5%, Na_3_VO_4 _0,2 mM, DTT 1 mM, PMSF 1 mM) in the presence of protease inhibitors (Complete, Boehringer) and incubated on ice. Cell debris were pelleted by centrifugation. Protein concentration was determined by Bradford (Biorad). Samples were loaded into 10% Tris/Glycine gels (Invitrogen) subjected to electrophoresis at 130V and transferred onto a nitrocellulose membrane (Immobilon-P, Millipore). Membranes were blocked in a 5% PBS-milk solution, incubated with a specific anti-GFP antibody (JL-8, BD 1:1000) overnight at 4°C. The next day, the membranes were washed and incubated with an anti-mouse horseradish peroxidase-conjugated secondary antibody (Amersham Biosciences 1:40000) and developed using the SuperSignal West Pico Kit (Pierce). To control for the amount of protein loaded per well, membranes were stripped with the Re-blot Plus Kit (Chemicon International), and re-probed with a specific anti β-tubulin antibody (sc9104 Santa Cruz Biotechnology 1:1000).

### Green fluorescent protein analyses

Twenty-four hours after transfection, the cells were washed with PBS, fixed with 4% paraformaldehyde (Sigma) and washed with PBS. Nucleic acids were stained with 4'-6'-diamine-2 phenylindole dihydrochloride (DAPI)-containing mounting medium (Vectashield, Vector). Cells were visualized with a Zeiss Axioplan 2 imaging microscope X40 using a Zeiss Axiocam HRc (color) camera and the Zeiss Apotome software. Given the fact that the localization of the GFP-fusion proteins is similar in Hela and in 293T, and because 293T cells are complex to handle in immunofluorescence experiments, we used these cells only for the western-blot analyses.

### Nuclear and cytoplasmic extraction

Twenty-four hours after transfection, the cells were washed with PBS. Nuclear and cytoplasmic fractions were then isolated using the sub-cellular proteome extraction kit (Calbiochem) following the manufacturer's instructions. Samples were subjected to immunoblot analyses as described above.

### NetNES software analysis

This software predicts leucine-rich nuclear export signals (NES) in eukaryotic proteins using a combination of neural networks (NN) and hidden Markov models (HMM). The prediction server calculates a combined 'NES score' from the NN and HMM scores. If the calculated 'NES score' exceeds the threshold, then that particular residue is expected to participate in a nuclear export signal. This is denoted with a "Yes" in the column "Predicted". Of note, the reason why one gets different scores for the same residues when comparing Tax1 and Tax2 sequences is that the score depends not only on the residue in question, but also on a number of previous residues which, in the case of E193 for example, are not identical between the two sequences.

## Competing interests

The author(s) declare that they have no competing interests.

## Authors' contributions

SAC, SC and LM performed the laboratory work. AG was involved in drafting the manuscript. LK participated in the interpretation of the NetNES server results and helped drafting the manuscript. RM designed, implemented and coordinated the study and wrote the manuscript. All authors have read and approved the manuscript.
